# Small RNA datasets of drug-susceptible *Mycobacterium tuberculosis* strains from Sabah, Malaysia

**DOI:** 10.1016/j.dib.2022.108795

**Published:** 2022-11-29

**Authors:** Mohd Iskandar Jumat, Jaeyres Jani, Zainal Arifin Mustapha, Kenneth Francis Rodrigues, Nornazirah Azizan, Armando Acosta, Maria E. Sarmiento, Kai Ling Chin

**Affiliations:** a Faculty of Medicine and Health Sciences, Universiti Malaysia Sabah, 88400, Kota Kinabalu, Sabah, Malaysia; b Borneo Medical and Health Research Centre, Faculty of Medicine and Health Sciences, Universiti Malaysia Sabah, 88400, Kota Kinabalu, Sabah, Malaysia; c Biotechnology Research Institute, Universiti Malaysia Sabah, 88400, Kota Kinabalu, Sabah, Malaysia; d School of Health Sciences, Health Campus, Universiti Sains Malaysia, 16150, Kubang Kerian, Kelantan, Malaysia

**Keywords:** Small RNA, RNA sequencing, *Mycobacterium tuberculosis*, Drug-susceptible, Sabah

## Abstract

These datasets present a list of small RNAs from three drug-susceptible *Mycobacterium tuberculosis* strains isolated from Sabah, Malaysia. Sputum samples were obtained from three tuberculosis patients belonging to different districts. The bacteria were detected using GeneXpert MTB/RIF, isolated and cultured in BACTEC^TM^ MGIT^TM^ 320, and tested for their drug susceptibility. Total RNAs were extracted, sequenced, and analyzed using bioinformatic tools to filter out small RNA present in the *Mycobacterium tuberculosis* strains. Small RNA sequencing generated total raw reads of 63,252,209, 63,636,812, and 61,148,224 and total trimmed reads (15-30 nucleotides) of 51,533,188, 53,520,197, and 51,363,772 for *Mycobacterium tuberculosis* strain SBH49, SBH149, and SBH372, respectively. The raw data were submitted to the Sequence Read Archive (SRA) database of the National Center for Biotechnology Information (NCBI) under the accession numbers of SRX16744291 (SBH49), SRX16744292 (SBH149), and SRX16744293 (SBH372). Small RNAs play important roles in cellular processes such as cell differentiation, cell signaling, development of resistance to antibiotics and immune response, and metabolism regulation. The small RNAs determined here could provide further insights into various cellular processes crucial for *Mycobacterium tuberculosis* survivability and a better understanding of their gene regulation which ultimately opens a new pathway for combating tuberculosis infection.

**Specifications Table**

**Table:** 

Subject	Molecular biology
Specific subject area	Transcriptomics
Type of data	Small RNA-sequencing datasets of three *Mycobacterium tuberculosis* strains, i.e., SBH49, SBH149, and SBH372, susceptible to streptomycin, isoniazid, rifampicin, and ethambutol.
How data were acquired	Illumina HiSeq 2500 sequencing platform
Data format	Raw data (fastq)
Description of data collection	The bacteria were isolated from sputum samples and cultured in Middlebrook 7H9 broth using BACTEC^TM^ MGIT^TM^ 320. Total RNAs were extracted from the pure culture and subjected to small RNA sequencing.
Data source location	• Region: Southeast Asia • State: Sabah • Country: Malaysia • District: Kota Kinabalu (SBH49), Penampang (SBH149), and Semporna (SBH372)
Data accessibility	Repository name: NCBI Sequence Read Archive Data identification number: SRX16744291 (SBH49), SRX16744292 (SBH149), SRX16744293 (SBH372) Direct URL to data: https://www.ncbi.nlm.nih.gov/sra/PRJNA863377

## Value of the Data

• The data provide valuable information for researchers involved in the studies of the relation of small RNAs with cellular processes such as cell signaling, and metabolism, among others, in different experimental conditions, and the discovery of novel small RNAs.
• The data are useful for researchers related to the study of the mechanisms of drug resistance, which could contribute to the establishment of biosignatures related to drug-susceptible and drug-resistant *Mycobacterium tuberculosis* strains.
• The data could provide valuable information to researchers looking for pathways useful for the development of new diagnostics, vaccines, and therapies for tuberculosis.
• The data will help to understand the genetic and functional characteristics of circulating strains in different geographical areas.

## 1 Data Description

Table 1 shows the pre-analysis data of raw reads for each sample obtained after sequencing. Initial raw reads of samples SBH49, SBH149, and SBH 372 were 63,252,209, 63,636,812, and 61,148,224, respectively. After trimming the adapter sequence and removing low-quality reads, a total of 11,719,021, 10,116,615, and 9,784,452 reads have been discarded from the samples SBH49, SBH149, and SBH372, respectively. The reads with 15-30 nucleotide (nt) of samples SBH49, SBH149, and SBH372 were 51,533,188, 53,520,197, and 51,363,772, respectively. A total of 11,676,526, 9,737,625 and 9,338,872 unique sequence tags have been determined for the samples SBH49, SBH149, and SBH372, respectively.

**Table 1:** Pre-analysis of raw reads.

ID	Raw reads	Trimmed reads (15-30 nt)	Discarded reads	Sequence tags
SBH49	63,252,209	51,533,188	11,719,021	11,676,526
SBH149	63,636,812	53,520,197	10,116,615	9,737,625
SBH372	61,148,224	51,363,772	9,784,452	9,338,872

**Fig. 1:**
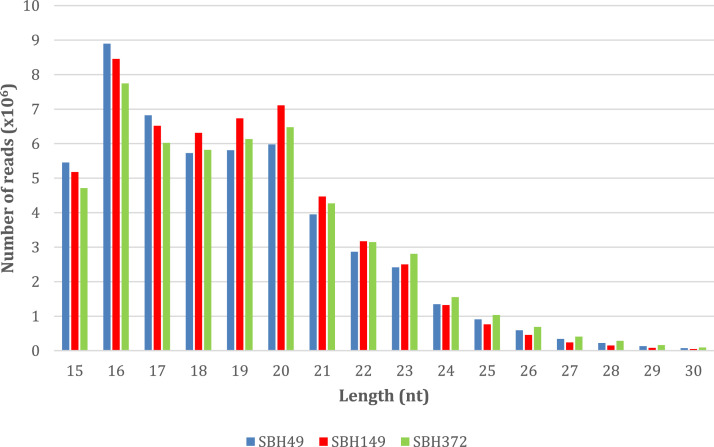
Length distributions of small RNAs.

Fig. 1 shows the length distribution of reads for each sample ranging from 15-30 nt. Read length with 16 nt was found to be abundant from the samples SBH49, SBH149, and SBH372 with 8,896,860, 8,454,546, and 7,746,989 reads, respectively.

**Fig. 2:**
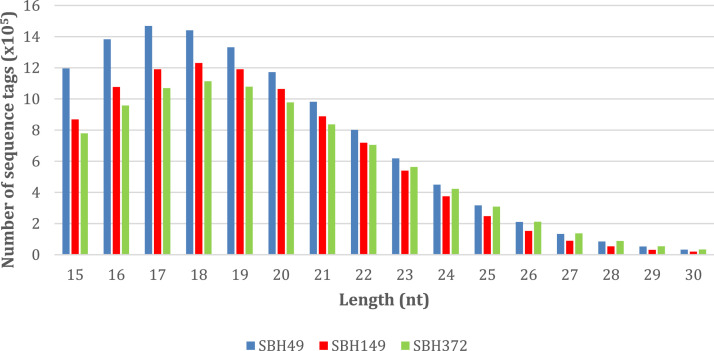
Length distribution of sequence tags.

Fig. 2 shows the length distribution of sequence tags for each sample. Read length with 17 nt was found to be abundant for sample SBH49 with 1,468,756 sequence tags, while read length with 18 nt was abundant for samples SBH149 and SBH 372 with 1,230,660 and 1,113,504 sequence tags, respectively.

## 2 Experimental Design, Materials and Methods

### 2.1 Culturing of *Mycobacterium tuberculosis*

*Mycobacterium tuberculosis* strains were isolated from three individuals who were diagnosed with pulmonary tuberculosis in the year 2018 from Kota Kinabalu (SBH49), Penampang (SBH149), and Semporna (SBH372) districts, respectively. Briefly, sputum samples were collected from individuals who presented with tuberculosis symptoms and abnormal chest X-rays. The sputum samples (0.5 mL) were analyzed with GeneXpert MTB/RIF (Cepheid, Sunnyvale, CA, USA) according to the manufacturer's protocol [1]. The bacteria present in the GeneXpert-positive samples were isolated using liquid culture media. First, the sputum samples were digested and decontaminated with mucolytic agent, BBL® MycoPrep™, to reduce the sputum viscosity and minimize contamination by rapidly growing normal flora. Then, each processed sputum was cultured in a tube containing Middlebrook 7H9 medium (7 mL) with BACTEC^TM^ MGIT^TM^ Growth Supplement (Becton, Dickinson and Company, USA) and BBL^TM^ MGIT^TM^ PANTA^TM^ (Becton, Dickinson and Company, USA) using BD BACTEC^TM^ MGIT^TM^ 320 system (Becton, Dickinson and Company, USA) at 37°C until the instrument signals the tube positive to growth. The bacteria culture were subjected to antibiotic susceptibility testing against streptomycin, isoniazid, rifampicin, and ethambutol at a final concentration of 1.0, 0.1, 1.0, and 5.0 µg/mL, respectively, using BACTEC^TM^ MGIT^TM^ SIRE kit (Becton, Dickinson and Company, USA) [2]. The isolates were preserved in 15% glycerol and stored at -80°C in the Microbial Culture Collection at the Faculty of Medicine and Health Sciences, Universiti Malaysia Sabah.

### 2.2 RNA extraction, quality control, and library preparation

Total RNA was extracted using Lucigen Masterpure^TM^ Complete DNA and RNA Purification kit (Epicentre Biotechnologies, Madison, WL, USA) with several modifications in the lysis method. Briefly, bacteria were revived from glycerol stock by culturing in a 7 mL MGIT for 14 days, the log phase for Mycobacterium growth. The bacteria in the tube were pelleted by centrifugation at 5,000 x g for 5 minutes. The supernatant was decanted and the cell pellet was mixed with 1X Tissue and cell lysis solution (600 µL) and transferred to a 1.5 mL Eppendorf tube. The cell mixture was transferred to a MN Bead Tube Type B (Macherey-Nagel, Düren, Germany) and was lysed with alternate bead beating for 1-minute and cooling on ice for 1-minute for a total of 20 minutes, followed by proteinase K (20 mg/mL) (40 µL) (Pygene^TM^, USA) treatment at 55°C for 15 minutes [3,4]. The remaining steps to obtain the total RNA pellet was conducted according to the manufacturer's instructions [5]. The pellet was resuspended in nuclease-free water (25 µL) and was analyzed with 1.5% agarose gel electrophoresis at 90V for 45 minutes using a pre-stained gel with FloroSafe Stain (First BASE Laboratories, Malaysia) and visualized using Gel Doc^TM^ XR (Bio-Rad, USA). The purity (A_260_/A_230_ and A_260_/A_280_) and concentration of the extracted RNA were measured by DS-11 spectrophotometer (DeNovix Inc, USA).

The RNA was outsourced for sample preparation and sequencing by Apical Scientific Sdn. Bhd, Malaysia. The integrity of RNA was determined by Agilent RNA 6000 Nano kit (Agilent Technologies, USA) [6]. Small RNA libraries preparation was carried out using NEBNext® Multiplex Small RNA Library Prep Set for Illumina® (New England Biolabs, UK), following the manufacturer's instructions. To prevent the production of dimers, the RNA samples were first ligated to the 3′ SR adaptor and primer hybridization was performed. Then, 5′ SR adaptor ligation and cDNA synthesis were performed. The small RNA libraries were enhanced via PCR amplification using a common primer and a primer containing one of the 48 index sequences. The libraries were gel purified by BluePippin^TM^ (Sage Science, USA). The libraries were quantified using KAPA Library Quantification kits for Illumina Sequencing platforms according to the qPCR Quantification Protocol Guide. Indexed libraries were pooled in equimolar amounts and sequenced on an Illumina HiSeq2500 instrument to generate 51-base reads.

### 2.3 Raw read pre-analysis and annotation

The data have been deposited in the NCBI (National Center for Biotechnology Information) [7] Sequence Read Archive (SRA) [BioSample accession number SRX16744291 (SBH49), SRX16744292 (SBH149), and SRX16744293 (SBH372)] under BioProject accession number PRJNA863377 (https://www.ncbi.nlm.nih.gov/sra/PRJNA863377) [8]. Further analysis was carried out using fastq-mcf tools version 1.04.676 [9]. The quality phred score used was Q20. Trimming of adapter index sequences was carried out and low-quality reads were removed to produce clean reads [9]. Clean reads having similar sequences were merged using R software and the redundant, partial sequences were removed to generate the sequence tags, the individual sequence which is specifically unique to a sRNA [10].

## Ethics Statements

The ethics approval for this study was obtained from Universiti Malaysia Sabah Medical Research and Ethics Committee [JKEtika 1/20 (10)]. Informed consent for sample collection was obtained from all the participants in this study. The authors kept the ethical concerns into consideration when gathering data and ensured that the information obtained from the respondents were only utilized for research purposes.

## CRediT authorship contribution statement

**Mohd Iskandar Jumat:** Writing – original draft, Investigation, Software, Data curation. **Jaeyres Jani:** Visualization, Investigation, Data curation. **Zainal Arifin Mustapha:** Supervision. **Kenneth Francis Rodrigues:** Supervision. **Nornazirah Azizan:** Supervision. **Armando Acosta:** Writing – review & editing. **Maria E. Sarmiento:** Writing – review & editing. **Kai Ling Chin:** Conceptualization, Supervision, Writing – review & editing.

## Declaration of Competing Interest

The authors declare that they have no known competing financial interests or personal relationships that could have appeared to influence the work reported in this paper.

## Data Availability

Small RNA Mycobacterium tuberculosis raw sequence reads (Original data) (NCBI).

## References

[1] Hillemann D., Rusch-Gerdes S., Boehme C., Richter E. (2011). Rapid molecular detection of extrapulmonary tuberculosis by the automated GeneXpert MTB/RIF system. J. Clin. Microbiol..

[2] Scarparo C., Ricordi P., Ruggiero G., Piccoli P. (2004). Evaluation of the fully automated BACTEC MGIT 960 system for testing susceptibility of Mycobacterium tuberculosis to pyrazinamide, streptomycin, isoniazid, rifampin, and ethambutol and comparison with the radiometric BACTEC 460TB method. J. Clin. Microbiol..

[3] Oh T., Kang H., Nam Y., Kim Y., You E., Lee M., Cho S., Lee H.J. (2016). An effective method of RNA extraction from *Mycobacterium tuberculosis*. Annal. Clin. Microbiol..

[4] Mangan J.A., Sole K.M., Mitchison D.A., Butcher P.D. (1997). An effective method of RNA extraction from bacteria refractory to disruption, including mycobacteria. Nucleic. Acids. Res..

[5] Gao J., Du J., Shu W., Liu Y., Wang Y., Xue Z., Li L., Pang Y. (2021). Stepwise selection of mutation conferring fluroquinolone resistance: multisite MDR-TB cohort study. Eur. J. Clin. Microbiol. Infect. Dis..

[6] Fleige S., Pfaffl M.W. (2006). RNA integrity and the effect on the real-time qRT-PCR performance. Mol. Aspects Med..

[7] N.R. Coordinators (2016). Database resources of the National Center for Biotechnology Information. Nucleic. Acids. Res..

[8] NCBI (2022). Small RNA *Mycobacterium tuberculosis* raw sequence reads. Natl. Center Biotechnol. Inform..

[9] Aronesty E. (2013). Comparison of sequencing utility programs. Open Bioinform. J..

[10] Schmieder R., Lim Y.W., Rohwer F., Edwards R. (2010). TagCleaner: identification and removal of tag sequences from genomic and metagenomic datasets. BMC Bioinform..

